# Zinc involvement in opioid addiction and analgesia – should zinc supplementation be recommended for opioid-treated persons?

**DOI:** 10.1186/s13011-015-0025-2

**Published:** 2015-08-04

**Authors:** Diana Ciubotariu, Cristina Mihaela Ghiciuc, Cătălina Elena Lupușoru

**Affiliations:** Pharmacology Department, University of Medicine and Pharmacy “Gr. T. Popa” Iași, Iași, Romania

**Keywords:** Zinc, Opioid dependence, Addiction, Analgesia

## Abstract

**Introduction:**

Zinc chelators were shown to facilitate some opioid-withdrawal signs in animals. Zinc deficiency, which affects more than 15 % the world’s population, is also common among opioid consumers and opioid-treated animals exhibit misbalances of zinc distribution.

**Aim:**

The present study focuses on how zinc ions interfere with opioid dependence/addiction and analgesia, trying to preliminary discuss if zinc supplementation in opioid-users should be recommended in order to reduce the risk of addiction.

**Methods:**

All relevant literature was searched up to April 2015. The search was performed using the term “zinc” plus combinations of following terms: “opioid receptors”, “opioid” or representatives of this class, “addiction”, “dependence”, “analgesia”, and “pain”. Human, animal, *in vitro* studies and reviews were including.

**Results:**

Both human and animal studies revealed decreased serum zinc under opioid-administration conditions, attributed mainly to increased urinary elimination (humans) or redistribution (animals). Moreover, animal studies revealed decreased brain zinc levels in morphine-treated animals, with increased zinc hepatic levels, but also an enhancement of endogenous opioid system activity and a possible reduction of morphine withdrawal by zinc. In vitro studies revealed reduction of opioid ligands binding to receptors by zinc. However, the very few *in vivo* animal studies on opioid analgesia revealed controversial results, as zinc demonstrated clear analgesic effect, but zinc associated to opioids doesn’t result in a potentiation of the analgesic effect.

**Conclusion:**

Zinc dietary supplementation in patients treated with opioids for cancer-related chronic pain should be considered, due to the high incidence of zinc deficiency, also well-documented in opioid consumers. The low toxicity of orally-administered zinc also pleads for this idea. The main contra-argument to zinc administration in opioid-treated persons is related to the way zinc influences opioid-induced analgesia.

## Introduction

Zinc, the second most abundant trace element in the human body (after iron) [1], plays mainly catalytic and structural roles [2]; as a co-factor of Cu-Zn superoxide dismutase, it is important for the anti-oxidant defence [3]. Zinc finger proteins acting as transcription factors regulate gene expression [4]. Within the central nervous system (CNS), zinc acts at the post-synapse level and influences synaptic plasticity, hormone release, and nerve impulse transmission [5–7], regulates post-synaptic proteins [8] and has important roles in the formation and maintenance of the structure of the post-synaptic density, a proteins network connecting the neurotransmitter receptors to the intracellular signalling system and to the cytoskeleton [9, 10].

The brain is one of the organs with high Zn^2+^ concentration, as it contains approximately 1.5 % of the estimated 2 to 3 g of zinc in the human body. Brain-zinc is non-uniformly distributed: the olfactory bulb has the highest level, followed by the frontal and parietal cortices, the striatum and the hippocampus; the lowest levels are in the medulla and thoracic spinal cord [11]. In the hippocampus, Zn^2+^ concentrations can reach up to 0.3 mM in the mossy fiber boutons of neurons extending from the dentate gyrus to neurons in the hilar and CA3 fields [12]. Distribution vary with the age: in rats, at birth, cerebellum has the highest zinc level; within time, the hippocampus and the cortex levels increase [13, 14]. Neuronal Zn^2+^ is either bound to various proteins (over 90 %, with structural or catalytic roles and roles in transcription factors and related proteins) or free (mainly localized within the vesicles of zinc-dependent glutamatergic neurons) [15].

Severe deficiency is rare, but mild to moderate zinc deficiency is widespread. Zinc deficiency affects more than 15 % of world population and the prevalence of inadequate intake varies from 7.5 % in high-income regions to 30 % in South Asia [16, 17]. Zinc intake is lower than recommended in more than 75 % of pregnant women [18]. Zinc is a type 2 essential nutrient [19], as deficiency symptoms are rather nonspecific; response to repletion occurs very fast [20]. Deficiency determines growth retardation, male hypogonadism, delayed wound healing and cell-mediated immune dysfunction [21]. Reduced zinc is also associated with DNA damage, increased inflammatory status [22, 23] and represents a predisposing factor for malignant tumours development [24]. Some deficiency-associated conditions are partially explained considering zinc’s role as a co-factor of enzymatic systems involved in nucleic acids replication, protein synthesis, and anti-oxidant defence. Disorders mainly related with CNS impaired function (behavioural changes, depression, emotional instability, anxiety, aggresivity, irritability, socializing deficits, impaired memory and learning, neurosensory alterations, anorexia etc.) are frequently associated with zinc deficiency in both humans and animal models [25, 26]. It is debatable, at least in case of some of these conditions, if decreased zinc is a consequence or a cause. Low zinc levels have been reported in autism spectrum disorders, attention deficit hyperactivity, schizophrenia, and spinocerebellar ataxia type 2 [27], underlining its importance in brain functioning. Under moderate zinc deficient diet in rats, brain zinc is maintained within normal limits, due to increased uptake [28], following transporters adaptation: the expressions of metallothionein-I and ZnT-1 are decreased, and that of LIV-1 is increased [29]; even so, subclinical deficiency affects human brain function [30].

Zinc salts are relatively nontoxic, particularly if taken orally. Symptoms including nausea, vomiting, epigastric pain, lethargy, and fatigue appear ‘at extremely high intakes (almost 100 folds above the recommended dietary allowance – 15 mg elemental zinc daily). Doses of 100-300 mg zinc daily, which are high above recommended, determine induced copper deficiency, anaemia and neutropenia, immune dysfunction and increased low-density vs. high density lipoproteins ratio [31–33]. Zinc toxicity is also manifest at the CNS level, where zinc has an additional role in the death of seizures-damaged neurons [34]. In Alzheimer’s disease, zinc has been shown to facilitate beta-amyloid aggregation [35]. In Parkinson disease, excessive hair zinc levels [36] and increased zinc exposure [37] are reported, but serum zinc is decreased [38]; *substantia nigra* zinc levels were found to gradually increase in rat model of parkinsonism [39].

A study of Larson et al., [40], evidenced increased withdrawal jumpings, but with no influence on acute dependence in conditions of zinc-chelator administration in mice. This is in accordance with preliminary data of ours showing a reduction of morphine dependence in rats by zinc chloride (ZnCl_2_) (dose-dependent) [41]. A possible reasonable, yet partial explanation for these results can be given considering zinc ions effect of reducing μ-opioid agonists’ binding to receptors [42, 43].

Taking into consideration zinc ability to reduce morphine-dependence intensity and the enhancement of withdrawal manifestations by zinc-chelators, we have formulated the hypothesis that zinc supplementation may reduce the risk of addiction in cancer-patients treated with opioids for chronic pain. The idea is reinforced by the low levels of zinc evidenced in opioid users [44–47] and the low toxicity of zinc is also in favour of this. The main contra-augment is that zinc, in spite of having well-documented analgesic action [48–54], may reduce the opioid analgesic efficacy, due to affecting binding affinity to receptors.

In this context, the present study aims to document, on the basis of the existent literature, the effect of zinc and zinc misbalances on opioid addiction, dependence and analgesia, trying to evidence and discuss if zinc supplementation in opioid users may be (or not) beneficial, in order to reduce the risk and/or the severity of addiction.

## Methods

All relevant literature (Scopus, Medline, and ProQuest databases) was searched up to May 2015. The search was performed using the term “zinc” plus combinations of following terms: “opioid receptors”, opioid or representatives of this class, “addiction”, “analgesia”, “algesia” and “pain”.

We have included articles (clinical studies, animal studies, in vitro studies and reviews – full-text or abstracts) containing information about:

1. zinc homeostasis in opioid consumers and opioid-treated animals compared to opioid-free individuals;

2. 1. the influence determined by zinc ions or zinc deficiency on the intensity of opioid-binding to specific and non-specific receptors (in vitro studies);

2. 2. the influence determined by zinc ions or zinc deficiency on the activity level of endogenous opioidergic system (animals and human studies);

3. the influence determined by zinc administration and zinc deficiency in animal models of opioid dependence and on the intensity of human opioid-addiction;

4. the influence of zinc ions or zinc deficiency on human and animal pain perception under opioid-free and opioid administration conditions.

Notes to the editors or comments were not considered. Search-retrieved articles which did not contain information on the mentioned issues were not considered.

Results of the initially-retrieved articles suggested some further searching strategies, which were applied to collect more relevant information. Some other relevant findings retrieved by initial searches are also presented when considered relevant, even though provided data do not perfectly fit initially-searched information. At each results sub-chapter, such things are being detailed.

Information on zinc-containing transcription factors, zinc-containing enzymes or zinc-finger proteins involvement on the addictive process or opioids mechanism of action was not considered.

Zinc ions and zinc deficiency influence on neurotransmitters important for development and maintenance of the addictive process (such as dopamine, glutamate etc.) or other mechanisms involved in drug addiction are not included, giving the complexity of such a topic and the difficulty of data interpretation.

## Results

The initial search-results provided the below-mentioned number of articles: 1. zinc + opioid: 133 articles; 2. zinc + opioid + receptor: 57 articles; 3. zinc + pain: 531 articles; 4. zinc + analgesia: 28 articles; 5. zinc + algesia: 34 articles; 6. zinc + addiction + opioid: 6 articles; 7. zinc + dependence + opioid: 7 articles.

Upon consulting the retrieved articles, only few of the retrieved search results were found to contain the information. Some of the articles were retrieved following at least two of the search terms combinations.

Results of the initially-retrieved articles suggested some further searching strategies, which were applied to collect more relevant information (for instances, we have used the names of the substances belonging to opioids class).

When considered relevant for the topic, additional information from the displayed articles is also presented at the results section. For instance, information about copper (a metallic trace elements to which zinc manifest a certain degree of biological antagonism) and metallothioneins (a family of cysteine-rich proteins strongly involved in both zinc and copper metabolism [55]) levels following opioids administration are also presented, as well as the data on trace elements content of different opioid preparations. The number of articles considered relevant is summarised at the beginning of each sub-chapter.

### Zinc misbalances in conditions of opioid administration

There are many humans studies reporting low zinc levels due to alcohol consumption [56–61]; studies about zinc levels in case of nicotine addiction or smokers are scarce and reveal non-concordant data [62–65]. There are no studies on microelements status in case of psychostimulants or cannabis administration in particular.

### Human studies

Upon refining the articles retrieved by the initial searches, we have found five studies which evaluated zinc status in persons consuming opioids (controlled-users or meeting diagnosis for opioid addiction), all stating either reduced zinc levels or increased elimination of zinc. Results present the concentration of zinc status in different body fluids (serum, plasma, cerebrospinal fluid), before, during and after the treatment (immediately, long-term after), but not zinc level in different organs. The main findings and their relevance to the evaluated topic are presented and commented below. Additional data considered relevant (such as copper status and information regarding zinc presence in opioid preparation) are also revealed in the below paragraphs.

Low zinc serum concentrations were reported in heroin consumers upon admission for treatment, with a slight tendency to increase during detoxification, yet not reaching the levels in controls [44]. In patients diagnosed with heroin addiction, zinc deficit appears to be correlated with the abuse period in case of heroin addiction [45] and low zinc plasma is accompanied by low intraerythrocytic zinc and by increased plasma and intraerythrocytic copper [46]. In case drug-free patients with heroin-addiction documented in the past, zinc concentration in the cerebrospinal fluid (CSF) of is lower than in controls, but generally within normal limits [47]. It is worth mentioning that the heroin samples on the illegal market, when analysed for the various metals contents, were shown to present variable amounts of zinc, but these traces have little contribution to product’s toxicity and to the trace elements intake [66, 67] and they probably appear from the metallic container used in the processing/cutting stage [68]. During methadone detoxification treatment for opioid addiction, zinc elimination is strongly increased, while copper elimination is slightly, but significantly decreased [69].

One of the problems with human studies is that not all of them consider the type of opioid drug, or the possible association with other consumed drugs or co-existent pathology; also, most of them fail to evidence the correspondences between abuse period, addiction severity and decreased zinc status and only one study [45] states that there is an association between the period of heroin consumption and the severity of zinc deficit. One of the studies makes little separation between heroin users and patients with mixed addiction (heroin + other drugs of abuse) [46].

Another problem is that these studies do not evidence if zinc deficiency is an effect of drug-taking or an associated condition in opioid-users. Feeding habits are not being considered, while malnutrition is common among people consuming drugs of abuse or alcohol [70], especially in women [71]. Malnutrition partially explains reduced zinc levels in case ethanol-consumption [58, 59]. Decreased CSF zinc in drug-sober former heroin users (even if concentration is generally within normal limits) [47] pleads that zinc deficiency in opioid users is rather a consequence of the alimentation habits, but evidences of increased urinary zinc elimination in patients taking methadone [69] as substitution therapy suggests that opioid-administration also has a specific role in this. Clinical studies should be interpreted taken into consideration the association between decreased appetite and decreased zinc. Malnutrition can be regarded as a cause of zinc deficiency, but, on the other hand, low level of zinc represents a cause of the lack of appetite, as documented by animals studies presented below [72, 73].

Between zinc and copper there is a certain degree of antagonism at the level of biological actions [74]; it appears that zinc deficiency has better conditions to more evidently manifest in opioid consumers, as decreased zinc is accompanied by increased serum and intraerythrocytic copper in heroin users [46] and given that copper urinary elimination is decreased under methadone maintenance treatment [69].

### Animal studies

We have found four relevant researches on the influence determined by opiods administration on zinc status or metallothioneins levels in animals. Two of them refer to zinc levels following morphine administration in rodents; one of them considers mainly zinc and metallothioneins liver level and the nature of its mediation, the other considers zinc levels in several organs and serum zinc concentration. Some aspects regarding metallothionein levels following morphine administration (presented by one of the two mentioned articles and by another article from those displayed by the initial search) was considered relevant for our topic. The forth study that we have included takes into consideration the influence of a δ-opioid receptor’s specific agonist on zinc levels of different brain regions. Main findings are exposed below, and briefly commented.

Morphine decreases serum zinc and increases liver citosolic zinc and metallothionein levels in rats. While morphine’s effect on liver metallothionein level appears to be mediated mainly by morphine’s action at receptors level (being antagonized by naloxone), liver zinc accumulation does not recognize this mechanism, as naloxone does not alter it; glucocorticosteroids and β-adrenergic mediation seem to be at least partially responsible for morphine effect of increasing zinc liver levels, as their blockade (with RU486 and respectively labetalol) diminishes it [75].

Interestingly, in a study investigating the effect of escalating doses of morphine in mice, increased zinc serum concentration and decreased liver zinc level were found; the brain zinc level was found decreased, as well as lungs and kidneys zinc levels, while spleen, heart and muscles zinc levels were found increased [76].

δ-opioid receptor’s agonist also decrease brain zinc level: (D-Pen2, D-Pen5)enkephalin (δ agonist) intracerebroventricular administration decreases parietal cortex, hippocampus and striatum Zn^2+^ contents. The effect is time- and dose-dependent and it is antagonized by naloxone pre-treatment [11].

Animal studies evidenced decreased brain zinc levels under opioid-administration conditions, which is difficult to document in case of humans (human studies only considered CSF, where zinc was found decreased in sober patients previously diagnosed with heroin-addiction [45]) and showed that one possible cause for zinc deficiency under opioid-administrating conditions is its re-distribution, mainly to the liver [75].

Table 1 summarizes the results of both human and animal studies.

**Table 1:** A table with a synthesis of above-presented data referring to zinc misbalances in conditions of opioid administration is presented below

biological fluid or structure where zinc concentration was determined		zinc concentration evolution compared to controls and mechanisms or details (where available)	
serum or plasma		decreased	Sadlik J. et al., 2000 (humans – in case of heroin users; tendency to normalize upon detoxification treatment, but not reaching levels in control group) [44]
			Elnimr T. et al., 1999 (humans – correlated with the abuse period) [45]
			Ruiz Martinez M. et al., 1999 (humans – accompanied by increased copper) [46]
			Iyengar V. et al., 1994 (humans – due to increased renal elimination) [69]
		increased	Floriańczyk B., 2000 (mice, morphine) [77]
red blood cells		decreased	Ruiz Martinez M. et al., 1999 (humans) [46]
cerebrospinal fluid		decreased	Potkin S.G. et al., 1982 (humans) [47]
other tissues	brain	decreased	Floriańczyk B., 2000 (mice, morphine) [77]
			Gulya K. et al., 1991 (rats) (parietal cortex, hippocampus and striatum) – induced by the δ- agonist (D-Pen2, D-Pen5)enkephalin, antagonized by naloxon [11]
	liver	increased	Hidalgo J. et al., 1991 (rats, morphine) [75]
		decreased	Floriańczyk B., 2000 (mice, morphine) [77]
	lungs, kidney	decreased	Floriańczyk B., 2000 (mice, morphine) [77]
	heart, spleen, muscle	increased	Floriańczyk B., 2000 (mice, morphine) [77]

## Zinc ions actions and zinc deficit effects on opioid system

### Zinc ions actions and zinc deficit effects on opioid binding to receptors (in vitro studies)

A very important component of zinc actions on mechanisms involved in drugs addiction takes place at the level of the opioid receptors. There are four studies reporting the inhibition of both agonists (endogenous and exogenous) and antagonist’s affinity to opioid receptors and two studies proving the increase of naloxone binding to opioid receptors in conditions of zinc-deficiency (chelators-use or following zinc-deficient diet). Additional information regarding possible interactions between zinc or zinc deficiency and the affinity of opioids to the non-opioid specific σ-receptors is revealed by other two studies. Table 2 summarizes the results.

**Table 2:** A synthesis of how zinc ions modulate the interactions between opioid receptors ligands and their receptors is presented below

		type of ligand	endogenous / exogenous (for agonists)	name	receptor	effects	brain structure	concentration	reference
zinc ion actions		agonists	endogenous	enkephaline	δ-receptor specific agonist	reduced both affinity and number of binding sites		physiological concentration	Ogawa N. et al., 1985 [43]
			exogenous	[3H] DAGO [([Tyr-D-Ala-Gly-Methyl-Phe-Glyol]-enkephalin	μ-receptor	reduced		micromolar concentration	Tejwani G.A., Hanissian S.H. 1990 [42]
					μ-receptor	reduced			Fowler C.B. et al., 2004 [78]
				[3H] DSTLE ([Tyr-D-Ser-Gly-Phe-Leu-Thr]-enkephalin)	δ-receptor	slighly reduced		micromolar concentration	Tejwani G.A., Hanissian S.H. 1990 [42]
				3H-met-enkephalinamide (2-D-ala-5-L-methionine)	δ-receptor	reduced	hippocampus, the cerebral cortex and the basal ganglia of the (rat)	endogenous concentrations	Stengaard-Pedersen K., 1982 [79]
				[3H] EKC (ethylketocyclazocine)	κ-receptor	slighly reduced		micromolar concentration	Tejwani G.A., Hanissian S.H. 1990 [42]
				(+)-[3H]pentazocine	σ_2_-receptor	slighly reduced	hippocampus (rat)	milimolar concentration	Connor M.A., Chavkin C. 1992 [83]
		antagonist		[3H]naloxone	μ-receptor	reduced affinity, with no effect on the number of binding sites	hippocampus, cortex, midbrain, striatum (rat)	dose-dependent manner	Hanissian S.H., Tejwani G.A. 1990 [85]
reduced zinc concentration compared to physiologic conditions	metallothionein peptide 1 (specific zinc chelator)	agonists	exogenous	(+)-[3H]pentazocine	σ_2_-receptor	non-changed	hippocampus (rat)		Connor M.A., Chavkin C. 1992 [83]
	zinc deficiency effect compared to normally-fed animals	antagonist		[3H]naloxone	μ-receptor	increased	isolated brain membranes (rat)		Essatara M.B., 1984 [72]

Physiologic zinc concentration inhibits the binding of specific agonists to μ-receptors; δ and κ receptors were relatively insensitive to this type of inhibition [42]. However, micromolar zinc concentrations inhibit the binding of enkephalin (specific δ-agonist) to receptors [43] and zinc ions determine a total blockade of the stereospecific δ-agonist 3H-met-enkephalinamide (2-D-ala-5-L-methionine) receptor-binding in synaptic hippocampus, cerebral cortex and basal ganglia membranes in rats, at in vitro concentrations compatible with endogenous zinc level in the mentioned structures. One more research [77] proves the inhibition determined by zinc ions on ligand-binding to μ-receptors and determines the aminoacids in the receptor’s structure which are responsible for it. This δ-agonists binding inhibition involves a decrease in both receptor affinity and the number of binding sites [78] and is due to the interaction of zinc with essential SH-groups in the opiate receptor structure. From the mentioned brain sites, it is especially the hippocampus which has high zinc content, almost exclusively localized in the giant boutons of the mossy fibers [79].

The σ-receptors were considered opioid receptors, but pharmacological tests showed that they are activated by drugs (such as phencyclidine or haloperidol) completely unrelated to the opioids and σ_1_ receptor has no structural similarity to the opioid receptors [80]. However, many opioids act as σ-agonists, pentazocine being one of the most potent σ-receptors ligands [81]. Stimulation of the zinc-containing mossy fibers in the hilar hippocampus region decreases the specific binding of (+)-[3H]pentazocine to σ-sites, unaffected by metallothionein peptide 1 (a specific zinc chelator), suggesting that zinc deficit has little effect on signalling through this receptor type [82]. However, increasing the free zinc ions concentration is an essential stage of the opioid-signalling inhibition via σ_1_-receptors and μ-receptors interaction, as shown by very recent findings [83].

Zinc ions inhibit the specific binding of [3H]naloxone (μ-receptors antagonist) to receptors in the rats hippocampus, cortex, midbrain and striatum (dose-dependent manner). The half maximal inhibitory concentration (IC_50_) is close to 30 μM, similar to the physiological zinc concentration in to extracellular brain environment. Inhibition is due to a decrease in receptor affinity, the number of binding sites being un-affected and it is reverted by histidine, which most probably acts as a chelator, as histidine alone has no effect on receptor affinity or the binding sites number [84]. This is in accordance with the proof that the [3H]-naloxone (1nM) binding to isolated brain membranes is increased in zinc deficient animals compared to ad libitum fed controls, while the weight restricted animals exhibit intermediate values [72].

### zinc ions actions and zinc deficit effects on endogenous opioidergic system activity

We have found just three relevant animal studies (and no relevant human studies) on zinc ions and zinc deficiency effect on the activity degree of endogenous opioid system. If there are clear, concordant data revealed by in vitro studies indicating the reductions of opioid binding to receptors by zinc, there are studies revealing that zinc determines important increases in the degree of activity of endogenous opioid system, while zinc deficiency determines opposite effects.

Acute third ventricle administration of zinc in minute amounts (3 to 300 pmol) induces a dose-dependent antidipsogenic action in dehydrated rats, which is almost complete at the highest dose. The effect is reversed by naloxone, indicating that it is at least partially due to stimulation of central opioid peptides [85].

Zinc deficient animals have reduced dynorphin (a leucine-enkephalin-containing opiate peptide and a potent inducer of spontaneous feeding) levels in the hypothalamus compared to *ad libidum*-fed animals, while weight-restricted animals exhibit intermediate values. Zinc-deficient animals are also relatively resistant to dynorphin-induced feeding. This suggests that zinc deficiency-induced anorexia is mediated by appetite deregulations of the endogenous opiate system, which appear to include alterations in receptor affinity, a post-receptor defect and alterations in the dynorphin synthesis and/or release [72]. Zinc ions inhibit pro-opiomelanocortin neurons in the hypothalamic arcuate nucleus (vital anorexigenic neurons) [73]. Although this fact represents a further proof of zinc-deficiency induced anorexia and its opioid-mediated origin, it seems reasonable to consider it slightly in disagreement with the above-discussed two studies (pro-opiomelanocortin is the precursor of the main endogenous opioids, and if pro-opiomelanocortin neurons are inhibited by zinc, it can be inferred that zinc reduces the level of brain-acting opioids; the article did not evaluate the level of extracellular opioids following zinc administration).

This proof of zinc deficiency-induced dysfunction of opioidergic system, together with the fact that zinc deficiency is common among opioid consumers, may constitute a supplementary explanation for a downward spiral: addiction and malnutrition are frequently associated; both of them are associated with zinc deficiency, which further contributes to anorexia, by reducing response to dynorphine-determined feeding.

### zinc ions actions and zinc deficit effects on opioid addiction or dependence

To our knowledge, there are no studies regarding zinc ions actions and zinc deficit effects on human opioid addiction or related conditions (tolerance, psychological dependence, craving etc.). There are, however, two animal studies focusing on zinc salts or zinc ions deficit on morphine dependence in rodents. Preliminary data of ours evidenced a reduction of morphine-induced dependence in rats by administration of ZnCl_2_, 0.1 and 0.05 mM/kg/day (intraperitonal), during the 10 days of morphine-dependence induction phase. The effect is dose-dependent and we consider that it can mainly be explained by reduced affinity of morphine to specific receptors in conditions of zinc excess. Morphine dependence intensity was evaluated by assessment of the naloxone-precipitated withdrawal [41]. Our results are in agreement with the study of Larson et al., [40], evidencing increased withdrawal jumpings (but with no influence on acute dependence) in conditions of zinc-chelator administration in mice.

## Zinc ions actions and zinc deficit effects on models of pain and on the opioid-mediated analgesia

### Human studies

Human studies about zinc influence on pain perception are rarely available or inconsistent. There are some data indicating the association between different forms of chronic pain and zinc deficiency, and a possible beneficial role of zinc supplementation in such cases, but overall, there is no uncountable prove of the analgesic effect of zinc in case of doses regularly used for oral supplementation. We have selected eight studies considered to have certain relevance to our topic and below we briefly present the data.

A clinical study revealed the association between low plasma zinc and tongue pain, a type of neuropathic pain [86]. Zinc levels in patients with trichodynia, a condition associating pain and a stinging sensation of the scalp related to diffuse alopecia, are not different compared to controls [87]. Zinc sulfate oral administration has better effects than placebo in alleviating dysmenorrhea [88] and reduces, up to total suppression, the muscle pain cramps in cirrhotic patients with hypozincemia [89]. The beneficial effect of a dietary supplement containing lycopene, epigallocatechin gallate, ellagic acid, selenium and zinc on chronic pelvic pain syndrome [90] and the reduction of chronic low back pain symptoms by alkaline minerals supplementation [91] were evidenced, but there is no proof of the specific role of zinc in neither of these. Zinc supplementation was thought to contribute to the prevention of sickle cell anaemia pain crisis, but it has not been scientifically proven [92, 93].

In brief, the results of the mentioned eight studies can be summaries as it follows: two of them claim the beneficial role of zinc administration in different forms of chronic pain [89, 90], two others claim that dietary supplements containing zinc (among others) alleviate certain forms of pain, but the particular role of zinc is not proven [91, 92]. One study reports low levels of zinc in a form of neuropathic pain [87], while another study reports that zinc levels is no different compared to control in another type of chronic pain [88]; two studies discuss the opportunity of zinc supplementation in sickle cell anaemia, but there is no clear proof of the role of zinc administration in preventing painful crisis specific to this disease [93, 94].

### Animal studies

Animal studies demonstrating the analgesic effects of non-toxic zinc doses are numerous and consistent; most of were performed in opioid-free animals. We have found six studies presenting zinc ions and/or zinc chelators effects on animal pain models in opioid-free conditions-only, five articles presenting zinc ions or zinc chelators effects on opioid-induced analgesia and one study evaluating the effect of zinc on pain under both opioid-free and opioid administrating conditions.

- zinc ions actions and zinc deficit effects on analgesia in opioid-free animals

One of the first evidences of the analgesic effects of zinc derives from the 1997 study on mice of Larson and Kitto [48], demonstrating a dose-dependent effect of intrathecal ZnCl_2_ in reducing the abdominal stretches and a strong effect of ZnCl_2_ (10 ng, intrathecal) in reducing capsaicin-induced biting and scratching behaviour (maximal after 90 min), but lack of effect on tail-flick latencies. Di-sodium calcium ethylenediamine tetra acetic acid (Na^+^Ca^2+^ EDTA) reduce ZnCl_2_ effect on writhing assay, and both Na^+^Ca^2+^ EDTA and dipicolinic acid (divalent cations chelators, the latter with greater selectivity for zinc) induce hyperalgesia in tail-flick assay, which is antagonized by zinc in a dose dependent manner in case of Na^+^Ca^2+^ EDTA.

Further studies of the same researchers (1999) indicated only a modest inhibition of writhing following intrathecal injection of high doses of zinc 24-h before writhing-induction, suggesting that, although zinc is necessary, but it may not be sufficient to induce a long-term antinociception. However, intrathecal co-administration of Na^+^Ca^2+^ EDTA (10-100 nmol) or dipicolinic acid (1 nmol) inhibited the antinociceptive effect of intrathecal capsaicine (2.8 nmol) or SP(1–7) (substance P) (10 nmol). None of the chelators alone influenced the writhing test in capsaicine- and SP(1–7)-free animals [49].

More evidences come from Liu et al., [50], revealing that intrathecal, intraplantar or systemic injection of ZnCl_2_ relieved thermal hyperalgesia in a dose-dependent manner in rats with sciatic nerve injury, while it has no influence on thermal nociception in the absence of nerve injury. Higher doses are required for intraperitoneal injections than for intrathecal or intraplantar injections [50].

The 2011 study of Matsunami M. et al. showed that the visceral nociceptive behaviour and abdominal allodynia/hyperalgesia caused by intracolonic sodium hydrosulfide in mice are abolished by intracolonic preadministration of ZnCl_2_ and reproduced by Zn^2+^ chelators. The effect of zinc in modulating this form of visceral pain is believed to be related to selective inhibition of T-type Ca^2+^ channels [51].

A very complex study of Nozaki et al. [52], revealed sustained zinc analgesia in different mice models of pain. Tail-flick latencies are increased following ZnCl_2_ intrathecal (0.2nM) and subcutaneous (0.1-1 mg/kg) administration, the effect being maximal at 90 min. In a model of inflammatory pain, ZnCl_2_ (0.2nM, intrathecal and 1 mg/kg, subcutaneous) determined strong antinociceptive effects, in both thermal and mechanical hyperalgesia. ZnCl_2_ (0.2nM, intrathecal and 0.1-1 mg/kg, subcutaneous) determined antinociceptive effects in a model of neuropathic pain induced by sciatic nerve ligation. It was demonstrated that zinc-induced analgesia is dependent on high-affinity binding of zinc to the NMDA receptor NR2A subunit, as it was completely abolished in NR2A-H128S knock-in mice, where zinc inhibition of NMDA currents was lost in the hippocampus and spinal cord under both acute and chronic pain conditions [52]. The study represents the first consistent evidence for the mechanism of zinc-determined analgesia, a well and long-time evidenced phenomena, which was previously believed to be related to zinc ability to stabilize primary afferent C-fibers.

Both zinc sulfate (0.5 and 2 mg/kg) and zinc citrate (0.125 and 0.5 mg/kg) (intraperitoneal) induced pain suppression in hot-plate and tail-flick tests (up to 17 %) and capsaicin writhing test (up to 25 %) in Swiss mice [53].

Hot plate assay show the analgesic effect of zinc oxide – conventional formulation (cZnO), 10 mg/kg, intraperitoneal, maximal at 90 min after administration. Zinc oxide – nanoparticals formulation (nZnO) determined higher analgesic effects compared to the conventional one, pain suppression of the modern formulation being evident at smaller doses and exhibiting a lower latency [54]. The acute analgesic effect of both zinc oxide formulations is antagonized by naloxone, revealing the role of endogenous opioids in zinc-analgesia.

Apart from the direct-acting mechanism of zinc as a pain-reliever, its anti-inflammatory effect, correlated with the pro-inflammatory status determined by zinc-deficiency [94], should also be taken into account.

b. zinc ions actions and zinc deficit effects on analgesia in opioid-treated animals

If there are uncountable proofs of the analgesic effect determined by zinc ions, in case of the association of zinc with analgesic opioids, most of the results seem at least controversial. The relevant retrieved search results on the interaction between zinc and analgesic opioids on pain perception are presented below.

The first study revealing zinc interaction with opioid analgesic effect states that Zn^2+^ administered intrathecally inhibited morphine antinociception in a dose-related fashion, but it also inhibited the development of acute tolerance to morphine antinociception (5 h after 100 mg/kg of morphine). However, intrathecal injection of Na^+^Ca^2+^ EDTA, a chelator of divalent cations, had no effect on analgesia and acute tolerance [40].

A more recent study proves that the acute analgesic effect (hot-plate assay) of morphine, 6 mg/kg (intraperitoneal) is increased by ZnO nanoparticals (5 mg/kg, intraperitoneal, co-administered with morphine), but it is not influenced by the classical ZnO formulation (10 mg/kg, intraperitoneal) [54]. The acute analgesic effects of 5 mg/kg nZnO and of 10 mg/kg cZnO (intraperitoneal) are antagonized by naloxone, 1 mg/kg, intraperitoneal co-administration, when evaluated 60 min after co-administration [54].

Preliminary own studies show that ZnCl_2_ (0.05 and 0.1 mg/kg/day, intraperitoneal, 10 days, during morphine-dependence induction) does not alter the acute effect of a high morphine dose in tolerant rats [95].

Coordination compounds of methenkephalin with transition metals (copper, cobalt, nickel and zinc) were shown to be superior to morphine by the analgesic activity and to morphine and methenkephalin by the duration of the analgesic effect [96].

Zinc deficiency tends to lower morphine-analgesic potency in mice [97].

A very recent study shows the enhancement of tramadol analgesic effects evaluated by tail-flick and hot-plate assay by intraperitoneal zinc administration (increase of approximately 30 % in the average pain inhibition compared with tramadol-alone at the most effective dose, which was 75 mg/kg b.w. zinc) in Swiss Weber male mice [98].

## Discussions

We found only two experiments focusing on the effects of zinc ions or zinc deficit on opioid dependence and results are accordant: zinc reduces dependence intensity when administered during dependence induction phase, while zinc chelators increase withdrawal manifestations (jumpings). Both these experiments used morphine and evaluated the intensity of naloxone-precipitated withdrawal [40, 41]. To our knowledge, there are no clinical studies regarding zinc influence on any type of substance-addiction or other addiction-types.

One shortcoming of the mentioned studies (on rats and mice) in terms of predictability for human aspects of addiction is that they did not use self-administration models: the doses of morphine used for dependence induction and the moment of their administration was established by the experimenter, and this represents a considerable difference compared to the characteristics of addiction in humans, where drug-taking habits have a strong determination by social conditions. Animal models of drugs self-administration are more predictive to human patterns of drug-taking compared to dependence animal-models where the drug administration is performed by external factors (such as the experimenter) [99]. Even more, the predictability of such animal experimental studies to humans is questionable, in the context of our hypothesis, especially as zinc was given by intraperitoneal injection in rats [41], whereas zinc salts as dietary supplements in humans are administered orally.

Most probably, the result of cited researches [40, 41] can be, at least partially, be put in relation with zinc ions property of decreasing morphine-binding to receptors, as revealed by in vitro studies. There are no specific data showing decreased morphine receptors-binding by zinc ions, but decreased binding of a μ-specific agonist by zinc was demonstrated [42] and other in vitro studies [43, 78, 79] showed reduced opioids-binding (both agonists and antagonist) to receptors by zinc, evident even at physiological concentration. The μ-receptors type is particularly susceptible to this inhibition type, but in case of δ-receptors, the number of binding sites is also decreased, while the affinity of opioids such as pentazocine to the non-opioid receptor σ is un-affected by zinc deficit [83]. The μ-type of opioid receptors-mediated effects has the most important contribution to addiction development [100]. Apparently in disagreement with the effect of zinc of lowering opioids biological effects by decreasing their binding affinity to receptors (evidenced by in vitro studies) are the evidences from animal experiments showing a positive correlation between the activity of endogenous opioids system and zinc levels [72, 86].

The results of the mentioned animal studies showing reduced morphine withdrawal in zinc-administrating conditions and withdrawal enhancement by zinc chelators [40, 41] suggest that *a possible beneficial role of zinc supplementation in humans taking opioid treatment for chronic pain* in reducing the risk of addiction development or the severity of addiction and tolerance is worth testing. Indeed, the risk of developing addiction in these patients is low (below 10 %) [101], but tolerance to opioids represents an important problem.

The idea of zinc supplementation in patients with malignant tumours taking opioids for pain suppression is reinforced by human studies documenting reduced zinc levels in opioid-users [44–47, 69]. In these patients, zinc deficit and its consequences as an additional strain to disease or to its associated conditions, can easily be avoided if discovered on time. However, low levels of zinc are mainly documented for the persons where controlled-use (as recreational drugs of abuse) has turned to addiction, and not for patients treated with opioids for chronic pain or other medical purposes. Most of the above-mentioned studies focused only on microelements homeostasis, but not on the feeding-habits. Opioids are known to induce side effects on the gastrointestinal tract, such as constipation, anorexia, vomiting, gastro-oesophageal reflux, abdominal pain [102] which may explain the tendency to malnutrition in opioid-users. One study assessing the changes in nutritional habits of opiates dependent persons during of methadone maintenance treatment revealed that, before enrolling in the treatment, the intake of calcium and magnesium was low, whereas low zinc intake was seen only in the women group; after four years of treatment, low zinc intake was noticed only in the men group. Several minerals and vitamins (such as iron, vitamins B1, B2, C, niacin) low-intake levels were revealed both before and after treatment, but there are significant differences between genders [103]. Apart from the human studies showing the association between decreased zinc levels and opioid-consumption, there are animal studies evidencing reduced brain zinc level following opioid administration [77], while data regarding zinc serum in opioids-treated animals are controversial [75, 77]. So, it is not well-established if opioids induce zinc deficiency or just altered distribution, as, on the other hand, there are also evidences that mild zinc deficiency is not accompanied by reduced brain zinc in rodents, as adaptation of the transporters favours the brain uptake of zinc ions [28, 29].

Zinc deficiency, associated to the depletion of other essential nutrients, including minerals and vitamins, can be considered a consequence of malnutrition. Both cancer and pain are conditions associated with the tendency to deficient feeding [104, 105]. On the other hand, reduced zinc level was proven to determine impaired-feeding in animal models [72, 73], while in humans, zinc administration alone (50 mg elemental zinc/day) is believed to have a role in alleviating feeding-habits in anorexia nervosa, by decreasing the level of depression and anxiety [106]. Reduced zinc also affects taste perception [25].

When discussing the opportunity of zinc administration in cancer patients, it should not be disregarded that zinc deficiency represents a predisposing factor for malignant tumours development, as zinc depletion increases the inflammatory status, impairs the immune system functioning, antioxidant defence, and affects the DNA structure [107]. Zinc deficit is common among cancer patients, which are also more prone to develop infections. So, zinc has a beneficial role in infections prevention by stimulating the immune system [21]. In case of cancer therapy, side effects of antitumor drugs (such as vomiting, diarrhoea) represent further factors which can favour nutrients deficit.

Moreover, decreased zinc has a certain influence on other mechanisms involved in the addiction downward spiral, as it is associated with depression-like symptoms and anxiety in both humans and animal models [26, 27], which are known to induce drug-taking behaviour [108]. Animals studies showed reduced endogenous opioidergic system activity following zinc depletion [72, 85]. Normalizing zinc level in zinc-deficient patients taking opioids for chronic pain could have a contribution to the reduction of the drug-seeking behaviour and of the self-administered opioid doses by attenuating depressive symptoms and enhancing endogenous opioidergic system activity, which may lower the craving.

So, as suggested by animal studies, we have hypothesised in the current review that zinc administration as dietary supplement may reduce the risk of developing dependence in patients with cancer treated with opiates for chronic pain. Apart from the assumption above, in the previous paragraphs, further possible benefits of zinc supplementation in such persons are discussed. To summarize, increasing zinc intake in these patients not only corrects a metabolic misbalance commonly encountered in both cancer and opioid-use (which is most likely not just a consequence of feeding-habits), but it may also improve conditions such as tendency to malnutrition, depressive-symptoms, anxiety, susceptibility to DNA-damage and infections. Other clinical signs and symptoms which would be alleviated by repletion (to which response is believed to appear fast [20]) might include: skin lesions, impaired night vision, altered smell and taste perception, growth retardation, male hypogonadism, impaired wound healing, increased inflammatory status [21–26].

Zinc low toxicity represents one more fact pleading for zinc supplementation in opioid-users [31–33]: when taken orally, even doses tens of times higher than recommended daily allowance determine only non-severe manifestations, mainly digestive.

If in vitro study evidences that zinc inhibition of opioid-agonists binding to receptors could explain, at least in part, reduced opioid dependence under zinc-administration conditions and withdrawal potentiating by zinc-chelators in rodents, the same evidences also raise the problem of decreased opioid analgesia potency under zinc-administrating conditions. Therefore, if zinc co-administration with opioids would decrease the risk and the severity of dependence/addiction development versus opioid-only administration, than this might come with the price of zinc lowering the analgesic effect of opioids. In humans, opioid doses used for pain suppression are self-regulated, and if zinc reduces opioid action at receptor level, it seems logical to consider that opioid potency is also affected, which may lead to increasing opioid administered doses. So, the chance to reduce addiction intensity or susceptibility to addiction becomes debatable. On the other hand, dependence is mainly mediated by μ-receptors [109], while in analgesia δ and κ-opioid receptors are also involved, but ligands affinity to δ and κ receptors is less affected by zinc inhibition, as shown in vitro [42].

In order to have an idea of the extent to which zinc might affect opioid-analgesia, the medical literature was searched to document the effect of zinc on pain perception in opioid-free and opioid-administrating conditions. The search revealed scarce data derived from the human studies. On the other hand, in animals, zinc formulations determine, at non-toxic systemic or local doses, indubitable analgesic effects in different models of pain: acute visceral, mechanical and thermal pain, under regular, inflammatory and neuropathic conditions [48–54]. Zinc effects on chronic pain animal models are less studied. It is, however, questionable if zinc analgesic effect can be additive to opioid-induced pain-suppression. In animal models, potentiation of morphine antinociception by zinc was evidenced in the case of a nano-particals ZnO formulation, but classical formulation does not modify morphine antinociception [54], while systemic zinc administration enhances tramadol-induced analgesia [98]. Our study showed that ZnCl_2_ administration during dependence-development does not alter morphine analgesia in tolerant rats [96], but others proved the inhibition of acute morphine antinociception by intrathecal zinc, however accompanied by tolerance inhibition [40]. Another study claims that zinc deficiency decreases opioid-analgesia in mice [97]. To our knowledge, in humans, the effect of zinc on opioid-analgesia (either in non-dependence, tolerance or addictive condition) has not been studied. Zinc supplementation influence on the analgesic effect of an acute opiate dose in dependent/tolerant individuals therefore becomes therefore very difficult to predict. Further clinical studies are needed, as tolerance to opioid is common in patients treated with such substances, especially the tolerance to analgesia [101]. If indeed, there are several data pleading in the favour of our hypothesis regarding the opportunity of zinc supplementation in persons treated with opioid for chronic pain suppression (such as zinc low toxicity, reduced zinc levels following opioid use, depressive symptoms associated with zinc deficiency, zinc lowering morphine dependence documented in rats), the problem of zinc interferences with opioid analgesia remains controversial and seems to represent the main contra-argument to the hypothesis. In case of animal studies, the inhibition of acute tolerance to morphine analgesia by zinc [40] was proven in mice, but it is accompanied by analgesia inhibition. The very few studies on how zinc affects opioid analgesia in opioid-dependent animals have not considered self-administration models [40, 96], but dependence models where administration is performed by the experimenter, whereas in humans, analgesic opioid doses and the moment of their administration are supposed to be self-controlled. Another problem is the lack of correspondence between doses used in acute pain animal models, where zinc is administered parenteral, and zinc levels reached at action site in case of oral supplementation.

We suggest a controlled clinical trial comparing the evolution of opioid doses needed for pain-relief in cancer patients cohorts with adequate nutrition versus adequate nutrition associated with zinc supplementation. It is mandatory to have a survey on the nutrition, associated drug-therapy, and environmental conditions in case of patient groups, as all these can determine the lack or excess of essential nutrients (including vitamins, minerals, aminoacids etc.) and as there is a dynamic cooperation and complex interaction and between these nutrients.

We speculate that zinc supplementation would most probably be beneficial for patients with cancer taking opioids for pain therapy, where dependence is less likely to develop, rather than for people taking them in recreational purposes, where psychological addiction is more frequent and severe [110]. Zinc supplementation for patients receiving methadone or other long-acting opioids maintenance treatment for opioid addiction is also worth being considered, especially as zinc urinary elimination is increased in case of methadone treatment [69].

Zinc ions strongly influence lots of neurotransmitter systems, including dopaminergic, glutamatergic, serotoninergic system; all of these has certain roles in addiction development and addiction-related phenomena, but such influence is not discussed in this review, due to the complexity of such a subject and to the difficulty of data interpretation.

## Conclusions

The present review pleads that a possible beneficial role of zinc dietary supplementation in patients with malignancies treated with opiates for pain-suppression should be considered. The main arguments are: 1) reduction of morphine-dependence intensity in an model where zinc was administered in escalating during morphine induction phase and potentiation of some morphine withdrawal manifestations by the use of zinc chelators (evidenced by animal studies); 2) reduced zinc level in opioid users; 3) zinc own analgesic effects (well-evidenced on experimental rodents studies and, to a lesser extent, revealed by clinical studies); 4) the low toxicity of zinc salts for oral administration. The main contra-argument is related to the suspected zinc effect of lowering the opioids analgesic efficacy, due to affecting the analgesic binding to specific receptors.

## References

[1] Fraga CG (2005). Relevance, essentiality and toxicity of trace elements in human health. Mol Aspects Med.

[2] McCall KA, Huang C, Fierke CA (2000). Function and mechanism of zinc metalloenzymes. J Nutr.

[3] Osredkar J, Sustar N (2011). Copper and Zinc, Biological Role and Significance of Copper/Zinc Imbalance. J Clinic Toxicol.

[4] Laity JH, Lee BM, Wright PE (2001). Zinc finger proteins: new insights into structural and functional diversity. Curr Opin Struct Biol.

[5] Bitanihirwe BK, Cunningham MG (2009). Zinc: the brain’s dark horse. Synapse.

[6] Xie X, Smart TG (1994). Modulation of long-term potentiation in rat hippocampal pyramidal neurons by zinc. Pflugers Arch.

[7] Lu YM, Taverna FA, Tu R, Ackerley CA, Wang YT, Roder J (2000). Endogenous Zn(2+) is required for the induction of long-term potentiation at rat hippocampal mossy fiber-CA3 synapses. Synapse.

[8] Grabrucker AM (2014). A role for synaptic zinc in ProSAP/Shank PSD scaffold malformation in autism spectrum disorders. Dev Neurobiol.

[9] Jan HH, Chen IT, Tsai YY, Chang YC (2002). Structural role of zinc ions bound to postsynaptic densities. J Neurochem.

[10] Grabrucker AM, Knight MJ, Proepper C, Bockmann J, Joubert M, Rowan M (2011). Concerted action of zinc and ProSAP/Shank in synaptogenesis and synapse maturation. EMBO J.

[11] Gulya K, Kovács GL, Kása P (1991). Partial depletion of endogenous zinc level by (D-Pen2, D-Pen5)enkephalin in the rat brain. Life Sci.

[12] Frederickson CJ, Bush AI (2001). Synaptically released zinc: physiological functions and pathological effects. Biometals.

[13] Sawashita J, Takeda A, Okada S (1997). Change of zinc distribution in rat brain with increasing age. Brain Res Dev Brain Res.

[14] Qian J, Noebels JL (2005). Visualization of transmitter release with zinc fluorescence detection at the mouse hippocampal mossy fibre synapse. J Physiol.

[15] Vogt K, Mellor J, Tong G, Nicoll R (2000). The actions of synaptically released zinc at hippocampal mossy fiber synapses. Neuron.

[16] Wessells KR, Brown KH (2012). Estimating the global prevalence of zinc deficiency: results based on zinc availability in national food supplies and the prevalence of stunting. PLoS One.

[17] Caulfield LE, Zavaleta N, Shankar AH, Merialdi M (1998). Potential contribution of maternal zinc supplementation during pregnancy to maternal and child survival. Am. J. Clin. Nutr.

[18] Fischer Walker CL, Ezzati M, Black RE (2009). Global and regional child mortality and burden of disease attributable to zinc deficiency. Eur J Clin Nutr.

[19] King JC (2011). Zinc: an essential but elusive nutrient1,2,3. Am J Clin Nutr.

[20] Moran VH, Stammers AL, Medina MW, Patel S, Dykes F, Souverein OW (2012). The relationship between zinc intake and serum/plasma zinc concentration in children: a systematic review and dose-response meta-analysis. Nutrients.

[21] Livingstone C (2015). Zinc: Physiology, Deficiency, and Parenteral Nutrition. Nutr Clin Pract.

[22] Bonaventura P, Benedetti G, Albarède F, Miossec P. Zinc and its role in immunity and inflammation. Autoimmun Rev. 2014, doi:10.1016/j.autrev.2014.11.008. 10.1016/j.autrev.2014.11.008 25462582

[23] Wong CP, Rinaldi NA, Ho E. Zinc deficiency enhanced inflammatory response by increasing immune cell activation and inducing IL6 promoter demethylation. Mol Nutr Food Res. 2015, doi:10.1002/mnfr.201400761 10.1002/mnfr.201400761 PMC4425307 25656040

[24] Ho E (2004). Zinc deficiency, DNA damage and cancer risk. J Nutr Biochem.

[25] Hagmeyer S, Haderspeck JC, Grabrucker AM (2015). Behavioral impairments in animal models for zinc deficiency. Front Behav Neurosci..

[26] Prasad AS (2013). Discovery of human zinc deficiency: its impact on human health and disease. Adv Nutr.

[27] Pfaender S, Grabrucker AM (2014). Characterization of biometal profiles in neurological disorders. Metallomics.

[28] Chowanadisai W, Kelleher SL, Lönnerdal B (2005). Zinc deficiency is associated with increased brain zinc import and LIV-1 expression and decreased ZnT-1 expression in neonatal rats. J Nutr.

[29] Takeda A, Tamano H (2009). Insight into zinc signaling from dietary zinc deficiency. Brain Res Rev.

[30] Sandstead HH (2012). Subclinical zinc deficiency impairs human brain function. J Trace Elem Med Biol.

[31] Taneja SK, Mandal R, Girhotra S (2006). Long term excessive Zn-supplementation promotes metabolic syndrome-X in Wistar rats fed sucrose and fat rich semisynthetic diet. Indian J Exp Biol.

[32] Fosmire GJ (1990). Zinc toxicity. Am J Clin Nutr.

[33] Salzman MB, Smith EM, Koo CJ (2002). Excessive oral zinc supplementation. Pediatr Hematol Oncol.

[34] Cuajungco MP, Lees GJ (1997). Zinc metabolism in the brain: relevance to human neurodegenerative disorders. Neurobiol Dis.

[35] Watt NT, Whitehouse IJ, Hooper NM (2010). The role of zinc in Alzheimer’s disease. Int J Alzheimers Dis.

[36] Forte G, Alimonti A, Violante N, Di Gregorio M, Senofonte O, Petrucci F (2005). Calcium, copper, iron, magnesium, silicon and zinc content of hair in Parkinson’s disease. J Trace Elem Med Biol.

[37] Pals P, Van Everbroeck B, Grubben B, Viaene MK, Dom R, van der Linden C (2003). Case-control study of environmental risk factors for Parkinson’s disease in Belgium. Eur J Epidemiol.

[38] Zhao HW, Lin J, Wang XB, Cheng X, Wang JY, Hu BL (2013). Assessing plasma levels of selenium, copper, iron and zinc in patients of Parkinson’s disease. PLoS One.

[39] Tarohda T, Ishida Y, Kawai K, Yamamoto M, Amano R (2005). Regional distributions of manganese, iron, copper, and zinc in the brains of 6-hydroxydopamine-induced parkinsonian rats. Anal Bioanal Chem.

[40] Larson AA, Kovács KJ, Spartz AK (2000). Intrathecal Zn2+ attenuates morphine antinociception and the development of acute tolerance. Eur J Pharmacol.

[41] Ciubotariu D, Nechifor M. Zinc involvements in the brain. Rev Med Chir Soc Med Nat Iasi. 2007;111:981–5. 18389791

[42] Tejwani GA, Hanissian SH (1990). Modulation of mu, delta and kappa opioid receptors in rat brain by metal ions and histidine. Neuropharmacology.

[43] Ogawa N, Mizuno S, Fukushima M, Mori A (1985). Effects of guanine nucleotides, transition metals and temperature on enkephalin receptors of rat brain membranes. Peptides.

[44] Sadlik J, Pach J, Winnik L, Piekoszewski W (2000). Concentration of zinc, copper and magnesium in the serum of drug addicts. Przegl Lek.

[45] Elnimr T, Hashem A, Assar R (1996). Heroin dependence effects on some major and trace elements. Biol Trace Elem Res.

[46] Ruiz Martínez M, Gil Extremera B, Maldonado Martín A, Cantero-Hinojosa J, Moreno-Abadía V (1990). Trace elements in drug addicts. Klin Wochenschr.

[47] Potkin SG, Shore D, Torrey EF, Weinberger DR, Gillin JC, Henkin RI (1982). Cerebrospinal fluid zinc concentrations in ex-heroin addicts and patients with schizophrenia: some preliminary observations. Biol Psychiatry.

[48] Larson AA, Kitto KF (1997). Manipulations of zinc in the spinal cord, by intrathecal injection of zinc chloride, disodium-calcium-EDTA, or dipicolinic acid, alter nociceptive activity in mice. J Pharmacol Exp Ther.

[49] Larson AA, Kitto KF (1999). Chelation of zinc in the extracellular area of the spinal cord, using ethylenediaminetetraacetic acid disodium-calcium salt or dipicolinic acid, inhibits the antinociceptive effect of capsaicin in adult mice. J Pharmacol Exp Ther.

[50] Liu T, Walker JS, Tracey DJ (1999). Zinc alleviates thermal hyperalgesia due to partial nerve injury. Neuroreport.

[51] Matsunami M, Kirishi S, Okui T, Kawabata A (2011). Chelating luminal zinc mimics hydrogen sulfide-evoked colonic pain in mice: possible involvement of T-type calcium channels. Neuroscience.

[52] Nozaki C, Vergnano AM, Filliol D, Ouagazzal AM, Le Goff A, Carvalho S (2011). Zinc alleviates pain through high-affinity binding to the NMDA receptor NR2A subunit. Nat Neurosci.

[53] Tamba BI, Leon MM, Petreus T (2013). Common trace elements alleviate pain in an experimental mouse model. J Neurosci Res.

[54] Kesmati M, Torabi M (2014). Interaction between Analgesic Effect of Nano and Conventional size of Zinc Oxide and Opioidergic System Activity in Animal Model of Acute Pain. Basic Clin Neurosci.

[55] Freisinger E, Vašák M (2013). Cadmium in metallothioneins. Met Ions Life Sci.

[56] McClain CJ, Su LC (1983). Zinc deficiency in the alcoholic: a review. Alcohol Clin Exp Res.

[57] Valberg LS, Flanagan PR, Ghent CN, Chamberlain MJ (1985). Zinc absorption and leukocyte zinc in alcoholic and nonalcoholic cirrhosis. Dig Dis Sci.

[58] Dinsmore W, Callender ME, McMaster D, Todd SJ, Love AH (1985). Zinc absorption in alcoholics using zinc-65. Digestion.

[59] Zarski JP, Arnaud J, Dumolard L, Favier A, Rachail M (1985). Trace elements (zinc, copper, manganese) in alcoholic cirrhosis: effect of chronic alcoholism. Gastroenterol Clin Biol.

[60] Giroux E, Schechter PJ, Schoun J, Sjoerdsma A (1977). Reduced binding of added zinc in serum of patients with decompensated hepatic cirrhosis. Eur J Clin Invest.

[61] Mills PR, Fell GS, Bessent RG, Nelson LM, Russell RI (1983). A study of zinc metabolism in alcoholic cirrhosis. Clin Sci (Lond).

[62] Arnaud J, Touvier M, Galan P, Andriollo-Sanchez M, Ruffieux D, Roussel AM (2010). Determinants of serum zinc concentrations in a population of French middle-age subjects (SU.VI.MAX cohort). Eur J Clin Nutr.

[63] Schuhmacher M, Domingo JL, Corbella J (1994). Zinc and copper levels in serum and urine: relationship to biological, habitual and environmental factors. Sci Total Environ.

[64] Szyszko M, Czarnowski W (2006). Smoking influence on cadmium, lead, selenium and zinc level in placenta, cord blood and maternal blood of women at delivery from Gdansk region. Przegl Lek.

[65] Unkiewicz-Winiarczyk A, Bagniuk A, Gromysz-Kałkowska K, Szubartowska E (2009). Calcium, magnesium, iron, zinc and copper concentration in the hair of tobacco smokers. Biol Trace Elem Res.

[66] Infante F, Domínguez E, Trujillo D, Luna A (1999). Metal contamination in illicit samples of heroin. J Forensic Sci.

[67] Bora T, Merdivan M, Hamamci C (2002). Levels of trace and major elements in illicit heroin. J Forensic Sci.

[68] Chan KW, Tan GH, Wong RC (2013). Investigation of trace inorganic elements in street doses of heroin. Sci Justice.

[69] Iyengar V, Chou PP, Costantino AG, Cook CB (1994). Excessive urinary excretion of zinc in drug addicts: a preliminary study during methadone detoxification. J Trace Elem Electrolytes Health Dis.

[70] Santolaria-Fernández FJ, Gómez-Sirvent JL, González-Reimers CE, Batista-López JN, Jorge-Hernández JA, Rodríguez-Moreno F (1995). Nutritional assessment of drug addicts. Drug Alcohol Depend.

[71] Saeland M, Haugen M, Eriksen FL, Smehaugen A, Wandel M, Böhmer T (2009). Living as a drug addict in Oslo, Norway--a study focusing on nutrition and health. Public Health Nutr.

[72] Essatara MB, Morley JE, Levine AS, Elson MK, Shafer RB, McClain CJ (1984). The role of the endogenous opiates in zinc deficiency anorexia. Physiol Behav.

[73] Qiu J, Zhang C, Borgquist A, Nestor CC, Smith AW, Bosch MA (2014). Insulin excites anorexigenic proopiomelanocortin neurons via activation of canonical transient receptor potential channels. Cell Metab.

[74] Angelova MG, Petkova-Marinova TV, Pogorielov MV, Loboda AN, Nedkova-Kolarova VN, Bozhinova AN (2014). Trace Element Status (Iron, Zinc, Copper, Chromium, Cobalt, and Nickel) in Iron-Deficiency Anaemia of Children under 3 Years. Anemia.

[75] Hidalgo J, Giralt M, Garvey JS, Armario A (1991). Effect of morphine administration on rat liver metallothionein and zinc metabolism. J Pharmacol Exp Ther.

[76] Floriańczyk B (2000). Zinc level in selected tissues of ethanol and morphine intoxicated mice. Med Sci Monit.

[77] Fowler CB, Pogozheva ID, LeVine H, Mosberg HI (2004). Refinement of a homology model of the mu-opioid receptor using distance constraints from intrinsic and engineered zinc-binding sites. Biochemistry.

[78] Stengaard-Pedersen K (1982). Inhibition of enkephalin binding to opiate receptors by zinc ions: possible physiological importance in the brain. Acta Pharmacol Toxicol (Copenh).

[79] Sindreu C, Storm DR (2011). Modulation of neuronal signal transduction and memory formation by synaptic zinc. Front Behav Neurosci.

[80] Fontanilla D, Johannessen M, Hajipour AR, Cozzi NV, Jackson MB, Ruoho AE (2009). The Hallucinogen N,N-Dimethyltryptamine (DMT) Is an Endogenous Sigma-1 Receptor Regulator. Science (New York, N.Y.).

[81] Wu HE, Hong JS, Tseng LF (2007). Stereoselective action of (+)-morphine over (-)-morphine in attenuating the (-)-morphine-produced antinociception via the naloxone-sensitive sigma receptor in the mouse. Eur J Pharmacol.

[82] Connor MA, Chavkin C (1992). Ionic zinc may function as an endogenous ligand for the haloperidol-sensitive sigma 2 receptor in rat brain. Mol Pharmacol.

[83] Rodríguez-Muñoz M, Sánchez-Blázquez P, Herrero-Labrador R, Martínez-Murillo R, Merlos M, Vela JM (2015). The σ1 receptor engages the redox-regulated HINT1 protein to bring opioid analgesia under NMDA receptor negative control. Antioxid Redox Signal.

[84] Hanissian SH, Tejwani GA (1988). Histidine abolishes the inhibition by zinc of naloxone binding to opioid receptors in rat brain. Neuropharmacology.

[85] Fregoneze JB, Luz CP, Castro L, Oliveira P, Lima AK, Souza F (1999). Zinc and water intake in rats: investigation of adrenergic and opiatergic central mechanisms. Braz J Med Biol Res.

[86] Yoshida H, Tsuji K, Sakata T, Nakagawa A, Morita S (2010). Clinical study of tongue pain: Serum zinc, vitamin B12, folic acid, and copper concentrations, and systemic disease. Br J Oral Maxillofac Surg.

[87] Durusoy C, Ozenli Y, Adiguzel A, Budakoglu IY, Tugal O, Arikan S (2009). The role of psychological factors and serum zinc, folate and vitamin B12 levels in the aetiology of trichodynia: a case-control study. Clin Exp Dermatol.

[88] Kashefi F, Khajehei M, Tabatabaeichehr M, Alavinia M, Asili J (2014). Comparison of the effect of ginger and zinc sulfate on primary dysmenorrhea: a placebo-controlled randomized trial. Pain Manag Nurs.

[89] Kugelmas M (2000). Preliminary observation: oral zinc sulfate replacement is effective in treating muscle cramps in cirrhotic patients. J Am Coll Nutr.

[90] Lombardo F, Fiducia M, Lunghi R, Marchetti L, Palumbo A, Rizzo F (2012). Effects of a dietary supplement on chronic pelvic pain syndrome (Category IIIA), leucocytospermia and semen parameters. Andrologia.

[91] Vormann J, Worlitschek M, Goedecke T, Silver B (2001). Supplementation with alkaline minerals reduces symptoms in patients with chronic low back pain. J Trace Elem Med Biol.

[92] Temiye EO, Duke ES, Owolabi MA, Renner JK. Relationship between Painful Crisis and Serum Zinc Level in Children with Sickle Cell Anaemia. Anemia. 2011, 698586. doi:10.1155/2011/698586 10.1155/2011/698586 PMC3065914 21490764

[93] Prasad AS, Cossack ZT (1984). Zinc supplementation and growth in sickle cell disease. Ann Intern Med.

[94] Prasad AS: Zinc is an antioxidant and anti-inflammatory agent: Its role in human. Frontiers in Nutrition 2014;1:14 doi:10.3389/fnut.2014.00014. 10.3389/fnut.2014.00014 PMC4429650 25988117

[95] Ciubotariu D, Pascu Baican M, Nechifor M, Tartau L. P.1.c.010 Zinc does not affect morphine analgesia in morphine-dependent rats. European Neurospsychopharmacology. 2012, 22, Supplement 2:S172.

[96] Gromov LA, Serdiuk EA (1990). The analgesic activity of coordination compounds of methionine enkephalin with divalent metals. Farmakol Toksikol.

[97] Dursun N, Erenmemisoglu A, Suer C, Gogusten B. The effect of zinc deficiency on morphine antinociception. Res Commun Alcohol Subst Abuse. 1995;16:47–52. doi:19174,35400005190799.

[98] Alexa T, Marza A, Voloseniuc T, Tamba B. Enhanced analgesic effects of tramadol and common trace element coadministration in mice. Neurosci Res. 2015. doi:10.1002/jnr.23609 10.1002/jnr.23609 26078209

[99] Lynch WJ, Nicholson KL, Dance ME, Morgan RW, Foley PL (2010). Animal Models of Substance Abuse and Addiction: Implications for Science, Animal Welfare, and Society. Comparative Med.

[100] Kieffer BL, Gavériaux-Ruff C (2002). Exploring the opioid system by gene knockout. Prog Neurobiol.

[101] Højsted J, Sjøgren P (2007). Addiction to opioids in chronic pain patients: a literature review. Eur J Pain.

[102] Leppert W (2012). The impact of opioid analgesics on the gastrointestinal tract function and the current management possibilities. Contemp Oncol (Pozn).

[103] Kolarzyk E, Chrostek Maj J, Pach D, Janik A, Kwiatkowski J, Szurkowska M (2005). Assessment of daily nutrition ratios of opiate-dependent persons before and after 4 years of methadone maintenance treatment. Przegl Lek.

[104] Suzuki H, Asakawa A, Amitani H, Nakamura N, Inui A (2013). Cancer cachexia—pathophysiology and management. J Gastroenterol.

[105] Zanocchi M, Maero B, Nicola E, Martinelli E, Luppino A, Gonella M (2008). Chronic pain in a sample of nursing home residents: prevalence, characteristics, influence on quality of life (QoL). Arch Gerontol Geriatr.

[106] Katz RL, Keen CL, Litt IF, Hurley LS, Kellams-Harrison KM, Glader LJ (1987). Zinc deficiency in anorexia nervosa. J Adolesc Health Care.

[107] Dhawan DK, Chadha VD (2010). Zinc: a promising agent in dietary chemoprevention of cancer. Indian J Med Res.

[108] Koob GF, Le Moal M (2001). Drug addiction, dysregulation of reward, and allostasis. Neuropsychopharmacology.

[109] Gavériaux-Ruff C (2013). Opiate-induced analgesia: contributions from mu, delta and kappa opioid receptors mouse mutants. Curr Pharm Des.

[110] Ahmad AH, Ismail Z (2002). c-fos and its Consequences in Pain. Malays J Med Sci.

